# Multiscale Encoding of Electrocardiogram Signals with a Residual Network for the Detection of Atrial Fibrillation

**DOI:** 10.3390/bioengineering9090480

**Published:** 2022-09-16

**Authors:** Mona N. Alsaleem, Md Saiful Islam, Saad Al-Ahmadi, Adel Soudani

**Affiliations:** Computer Science Department, College of Computer and Information Sciences, King Saud University, Riyadh 11543, Saudi Arabia

**Keywords:** atrial fibrillation, deep learning, CNN architecture, residual network, multiscale

## Abstract

Atrial fibrillation (AF) is one of the most common cardiac arrhythmias, and it is an indication of high-risk factors for stroke, myocardial ischemia, and other malignant cardiovascular diseases. Most of the existing AF detection methods typically convert one-dimensional time-series electrocardiogram (ECG) signals into two-dimensional representations to train a deep and complex AF detection system, which results in heavy training computation and high implementation costs. In this paper, a multiscale signal encoding scheme is proposed to improve feature representation and detection performance without the need for using any transformation or handcrafted feature engineering techniques. The proposed scheme uses different kernel sizes to produce the encoded signal by using multiple streams that are passed into a one-dimensional sequence of blocks of a residual convolutional neural network (ResNet) to extract representative features from the input ECG signal. This also allows networks to grow in breadth rather than in depth, thus reducing the computing time by using the parallel processing capability of deep learning networks. We investigated the effects of the use of a different number of streams with different kernel sizes on the performance. Experiments were carried out for a performance evaluation using the publicly available PhysioNet CinC Challenge 2017 dataset. The proposed multiscale encoding scheme outperformed existing deep learning-based methods with an average F_1_ score of 98.54%, but with a lower network complexity.

## 1. Introduction

Cardiovascular diseases have been found to be the leading causes of morbidity and mortality in developed countries [1]. Atrial fibrillation (AF) is the most common type of cardiac arrhythmia associated with cardiovascular disease and is a symptom of an increased risk of stroke, heart failure, and coronary artery disease. Currently, AF affects 33.5 million people globally, and this figure is expected to increase rapidly due to the aging of the population [2]. It occurs due disorganized electrical activity in the upper left chamber of the heart (the left atrium), which makes the atria quiver or fibrillate [3].

Clinically, AF is characterized by two main features: the absence of P waves, which are replaced by a fast oscillation of fibrillatory waves, and the irregularity of the RR intervals, which is caused by uncoordinated electrical impulses that disrupt the normal activation of the atria. It usually attacks as paroxysmal AF, which typically happens outside the hospital and raises the need for fast and accurate detection of AF. Several established techniques based on ECG features, such as QRS complexes, RR intervals, and heart rate variability, have been in use to automatically recognize AF segments from the ECG signals [4,5]. Most of these techniques produce unsatisfactory results and low classification performance, which is mostly because of their failure to detect and extract the characteristic features precisely.

The use of advanced signal processing and machine learning techniques in AF detection can help to reduce the error rate while improving diagnosis accuracy and timeliness, as seen in many other fields, such as cancer diagnosis [6] and DNA analysis [7]. With traditional machine learning, the process of AF detection starts with the extraction of handcrafted features and continues with the use of these features to train a classification model. Several classifiers were utilized, including support vector machines (SVMs) [8,9,10], k-nearest neighbors [11,12], and an artificial neural network (ANN) classifier [13,14]. The detection or classification performance of these techniques was significantly affected by the quality of the extracted features. To overcome these challenges, many attempts were made to improve the detection of the features using deep learning (DL) techniques.

Deep-learning-based methods provide a significant advantage over traditional techniques, where feature extraction is performed automatically using the model’s powerful data learning capabilities. Several deep learning techniques were proposed and showed excellent performance, such as convolutional neural networks (CNNs) [15], recurrent neural networks [16], and autoencoders [17]. However, automatic learning of the characteristic features using DL techniques is still a challenging issue, and there are many attempts in the literature to enhance the performance of the deep networks, such as by using different preprocessing and transformation techniques, which might be computationally inefficient. Another attempt is the use of multi-scale fusion [18] of deep neural networks to improve the detected features, thus allowing the network to look at them at different scales rather than at a single scale.

The problem of selecting appropriate deep learning structures remains unresolved. There has been little progress in developing an appropriate neural network architecture that achieves high performance on medical data, specifically on AF detection using ECG signals. Furthermore, many of the structures proposed in the literature comprise hundreds of layers and millions of parameters, making the process of AF detection computationally heavier than it should be. In addition, most of the existing CNN-based methods employ a deep network with fixed-size kernels as the receptive field in the convolution operation. This may not aid the comprehensive capture of crucial features in ECG signals, since the use of the same kernel only allows the feature representation in a limited area of the input to be considered. Further attention to solving all of these problems is needed.

In this paper, a novel DL model is proposed, consisting of a multiscale encoding scheme and parallel one-dimensional (1D) sequences of residual blocks. The proposed multiscale encoding along with the residual blocks helps to extract diagnostic features from short single-lead normalized ECG signals using multiple streams of different kernel sizes, and these features are passed to dense layers for the classification of the ECG signal into two classes. The important contributions of this paper are as follows:

1. A multiscale encoding scheme using a parallel structure of 1D residual blocks with different kernel sizes for capturing features at different scales is proposed. This multiscale method looks at the signal from different perspectives, yielding a better representation of diagnostic features. This also allows networks to grow in breadth rather than in depth, thus reducing the computing time by using the parallel processing capability of deep learning networks.
2. We investigated the effects of the use of different numbers of streams and different kernel sizes on short single-lead normalized ECG signals and experimented with the effects on the detection performance.
3. We developed an efficient multiscale 1D CNN network that receives 1D ECG signals without any conversion or transformation and does not require explicit preprocessing or feature engineering.
4. This paper investigates the effects of different lengths of segments of ECG signals, different balancing techniques, and different parameters on the detection performance.

The remainder of this paper provides a literature review, an overview of our proposed multiscale encoding scheme with a description of our proposed DL model, and a detailed explanation of the steps taken to accomplish the AF classification goal. The results are discussed in detail in Section 5.

## 2. Related Works

During an AF rhythm, the electrical impulses in the atria come from diverse locations, rather than the typical sinoatrial node; they propagate quickly and erratically throughout the atria, resulting in a highly irregular ventricular rate. Clinically, AF can be detected by observing the ECG signal waveform and looking for specific features, such as a missing or absent clear P wave, irregular beat patterns, and abnormal RR intervals. Numerous efforts have been made by researchers around the world to automatically learn these features that define AF. Specially, the irregularity of RR intervals was characterized in numerous studies using different measures such as the entropy, standard deviation, and median; AF detection was performed by using feature thresholding and machine learning based techniques [19,20]. Liu et al. [21] used an SVM-based method to detect AF among four arrhythmias, achieving a moderate performance with an accuracy of 86.23%. However, the performance of these techniques is highly dependent on the detected features, where the handcrafted features are not typically robust for many variations, such as scaling, noise, and displacement. In addition, these methods suffer from the well-known issue known as overfitting, where the model fails to generalize well on unseen data.

Deep learning (DL) has recently been developed to address several issues with conventional techniques and has proven to be very effective in solving a variety of issues. Especially, CNNs, with their strong feature extraction capabilities, are becoming popular in various fields, including signal processing. Many researchers have attempted to use DL as a solution to the AF detection problem, as shown in Table 1 and Table 2. Pourbabaee et al. [22] used a CNN to develop an intelligent system where automatically learned AF features were used for the classification. Andreotti et al. [23] used a Residual Network (ResNet) [24], a variation of CNNs, to develop an AF detection system. Such networks can train deeper networks because of the residual connections that help solve the issue of vanishing gradients. Wang et al. [25] developed an 11-layer neural network structure that was primarily stacked with a CNN and a modified Elman neural network (MENN) for AF detection, which had an accuracy of 97.5% and a sensitivity of 97.9%. Limam et al. [26] proposed a convolutional recurrent neural network (CRNN) that comprised two independent CNNs for extracting features—one from ECGs and the other from heart rates. The CRNN structure consists of layers of CNNs and long short-term memory (LSTM). An AF detection method was proposed [16] based on an end-to-end 1D CNN architecture, which had data length normalization, training, and prediction phases. The proposed structure included a 10-layer architecture for classifying the signal into four classes, such as AF, normal, noise, and others. It achieved an average F_1_ score of 78.2%. Xiong et al. [16] proposed another DL approach that used a 16-layer 1D CNN network to classify AF from the other classes. This approach achieved 82% detection accuracy.

Despite the wide use of 1D CNNs in time-series classification, numerous studies have attempted to improve AF detection and classification accuracy by using new techniques with proven success in the computer vision domain. Such techniques transform the signals into a two-dimensional (2D) representation. In a previous study [27], we proposed a network of two layers of bidirectional LSTM for improving AF detection accuracy. Our approach started with the augmentation of the minor class for balancing labels and moved to the transformation of the segmented signals into a spectrogram using short-time Fourier transforms over time windows to extract two types of time–frequency (TF) moments—instantaneous frequency and spectral entropy—that were eventually fed to the LSTM for training and final classification. The approach was evaluated using the PhysioNet Challenge 2017 dataset [28] and was found to be 91.4% accurate. Zihlmann et al. [29] proposed a technique that trained a CRNN using an ECG signal’s spectrogram. This procedure first applied a quick Fourier transform on ECG data to create a 2D spectrogram. To aggregate features, the spectrogram was first input into a stack of 2D convolutional blocks and then into a three-layer bidirectional LSTM network. However, the complexity of the network was relatively high, with more than 10 million training parameters, which is a major drawback when using signal transformation. Furthermore, researchers [23,30] found that transformation from 1D signals to 2D images improved the classification accuracy by approximately 3%, but at the expense of higher conversion costs and more complicated networks.

Recently, a fusion of deep neural networks was proposed as another attempt to improve feature detection and classification. A fusion of CNNs was proposed in [21] that employed two-stream convolutional networks with different filter sizes to extract features of different scales. Yao et al. [31] proposed a multiscale convolutional neural network (MCNN) that applied time scaling on input signals and detected AF based on the scaled inputs. The authors deployed different signal transformation schemes, including identity mapping, down-sampling transformations in the time domain, and spectral transformations in the frequency domain. Each part was known as a branch, as it was a branch input for the CNN for final classification. The MCNNs showed improvements against the other methods in time-series classification [18].

Many of these existing methods were evaluated using landmark databases, such as the MIT Atrial Fibrillation Database (AFDB) [32], and achieved excellent AF detection accuracy (e.g., 99.19% sensitivity and 99.39% specificity in [33]). Existing methods seemed less promising when evaluated with other publicly available databases, such as the recently introduced PhysioNet Challenge 2017 dataset [28]. One practical issue that has been considered is the class imbalance problem, which has been established as affecting both convergences during the training phase and the generalization of a model in the test phase, with a significant detrimental effect on the classifier accuracy. Researchers have tried to solve the problem of imbalance distribution. For example, Acharya et al. [34] proposed synthetic data as a solution. Similarly, Jiang et al. [35] produced synthetic data, but they used the concept of oversampling the minority class data. Another method [36] used skewness to drive the dynamic data augmentation technique for balancing data distribution.

**Table 1:** State-of-the-art AF detection methods based on convolutional neural network (CNN) models with respect to the database, structure, classes, scales, transformation, balancing type, and performance.

Reference	Database	Structure	Classes	Scales	Transformation	Balancing	Performance
Pourbabaee et al. [22]	PAF DB Challenge 2001	CNN	2	Single	-	-	CE: 82%
Hsieh et al. [37]	PhyNetCha17 *	1D CNN	4	Single	-	-	F_1_: 78.2%
Xiong et al. [16]	PhyNetCha17 *	1D CNN	4	Single	Spectrogram	-	F_1_: 82%
Fan et al. [38]	PhyNetCha17 *	CNN	4	2	-	Replication	F_1_: 98.13%
Yao et al. [31]	MIT–BIH AF **, HEDB	CNN	2	3	Time-Domain Transformation	-	ACC: 98.18% SEN: 98.22% SP: 99.11%
Fan et al. [39]	PhyNetCha17 *	2D CNN	3	Single	-	Horizontal and Vertical Flip	F_1_: 87.22%
Xu et al. [40]	MIT–BIH AF **	2D CNN	2	Single	Wavelet Transform	-	ACC: 81.07%
Prabhakararao et al. [41]	PhyNetCha17 *	CNN	2	4	Spectrogram	-	F_1_: 84.31%
Ping et al. [42]	PhyNetCha17 *	CNN	2	3	-	Regular OS ^+^	F_1_: 89.55%

* PhysioNet Challenge 2017 (PhyNetCha17), ** MIT–BIH Atrial Fibrillation (MIT–BIH AF), ^+^ Oversampling (OS).

Table 1 and Table 2 summarize the state-of-the-art AF detection methods based on the database, structure, number of classes, number of scales, transformation technique, balancing type, and performance. Table 1 shows the CNN-based methods, and Table 2 shows methods utilizing hybrid models. The abovementioned studies showed that research on the analysis of ECG signals is mainly based on single-scale signal analysis techniques using conventional CNN frameworks that have been found to be efficient in obtaining representative features, but that lack in design optimization and parameter tuning. This challenge must be addressed without using rigorous preprocessing or data transformation for the extraction of the features to increase the detection accuracy without increasing the computational overhead or network complexity.

**Table 2:** State-of-the-art atrial fibrillation (AF) detection methods using hybrid models with respect to the database, structure, classes, scales, transformation, balancing type, and performance.

Reference	Database	Structure	Classes	Scales	Transformation	Balancing	Performance
Andreotti et al. [23]	PhyNetCha17 *	ResNet	4	Single	-	Regular OS ^+^	F_1_: 72%
Wang et al. [25]	MIT–BIH AF **	MENN, CNN	4	Single	-	-	ACC: 97.4% SE: 97.9%, SP: 97.1%
Limam et al. [26]	PhyNetCha17 *	CNN, RNN	4	Single	-	-	F_1_: 77%
Alsaleem et al. [27]	PhyNetCha17 *	BI–LSTM	2	Single	Spectrogram	Regular OS ^+^	ACC: 91.4%
Zihlmann et al. [29]	PhyNetCha17 *	CNN, LSTM	4	Single	Spectrogram	-	F_1_: 83%
Haddi et al. [33]	MIT–BIH AF **	PCA, LVQ	2	Single	-	-	SEN: 99.19% SP: 99.39%
Faust et al. [43]	MIT–BIH AF **	BI–LSTM	2	Single	-	-	ACC: 98.15%
Change et al. [44]	Several	LSTM	2	Single	Spectrogram	-	ACC: 87%
Maknickas et al. [45]	PhyNetCha17 *	LSTM	4	Single	-	-	F_1_: 78%
Schwab et al. [46]	PhyNetCha17 *	RNN	4	Single	-	Augmentation	F_1_: 79%
Andersen et al. [47]	AFDB, MITDB, NSRDB	CNN, RNN	2	Single	-	-	SE: 98.98% SP: 96.95%
Farhadi et al. [48]	MIT–BIH AF **	AE	2	Single	-	-	ACC: 93.60%
Xiong et al. [49]	PhyNetCha17 *	CNN, RNN	3	Single	-	-	F_1:_ 82%
Wang et al. [50]	MIT–BIH AF **	RNN	2	3	-	-	ACC: 98.3%

* PhysioNet Challenge 2017 (PhyNetCha17), ** MIT–BIH Atrial Fibrillation (MIT–BIH AF), ^+^ Oversampling (OS).

## 3. Multiscale Encoding of ECG Signals Using the ResNet

The majority of the existing works in the literature use a fixed-size single-scale kernel as the receptive field in the convolution operation, which might not fully capture crucial features of ECG signals. In this work, a deep architecture for AF detection has been created based on the multiscale convolution kernel in order to enhance the feature representations. Additionally, the residual network (ResNet), which has a stronger feature extraction capability, is used to manage the extracted features. Hence, we named our model for encoding ECG signals the multiscale residual network (MsRes), which aims to better represent the features suitable for automatic learning and classification.

The primary goal of our model is to classify short segments of ECG signals into AF or non-AF classes. The raw 1D time-series data are fed into the network without conversion or transformation. Additionally, since our AF detection is data-driven, no manual feature engineering methods are required to learn the features of the ECG signals, as they are learned directly from the input data. Our model has two main parts: the multiscale signal encoding part, which aims to improve the AF diagnostic feature detection, and the classification part, which aims to predict one of the two labels.

### 3.1. Multiscale Encoding

Suppose we have a segment of the ECG signal *f*(x). For multiscale encoding of the signal, first, the signal will convolve with different convolution kernels as follows:

(1) $$f_{i}^{\prime }\left(x\right)=f\left(x\right)\times h_{i}\left(x\right),$$

where *h_i_*(*x*) is a convolution kernel for the scale *i* = 1…*n*, and the size of the kernel is proportional to the scale.

**Figure 1:**
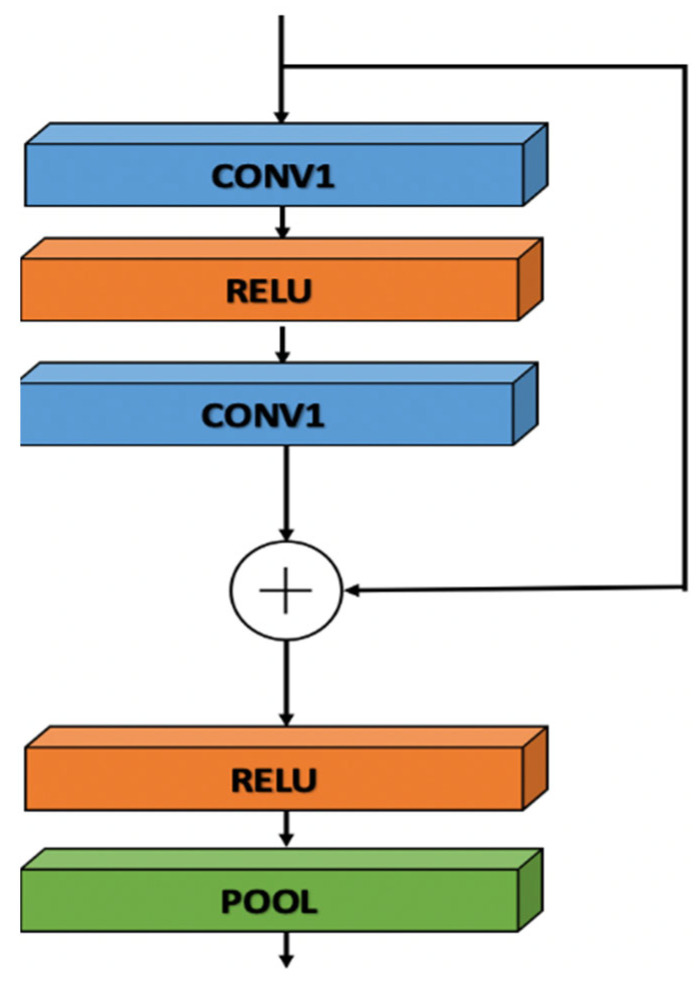
The residual block structure of the multiscale residual network (MsRes).

We utilized ResNet-based DL techniques for the multiscale encoding of the convoluted signal *f_i_*′(x). MsRes blocks were designed and built depending on experiences in ResNet, which outlined the idea of an identity shortcut link, which limited the volume of information that could pass through the shortcut [36]. By leveraging activations from earlier layers, ResNet overcomes the issue of disappearing and exploding gradients. The network is made simpler by the shortcut connection by employing fewer layers and less backpropagation (BP), which decreases the size of the model and speeds up execution. As shown in Figure 1, each residual block contains two convolutional layers and two rectified linear units (ReLUs) [51], which are well-known activation functions in convolutional networks. When the input is positive, the ReLU function is less susceptible to the gradient saturation issue than the sigmoid and tanh functions are. Additionally, each residual block comprises a residual skip connection [24] and a pooling layer that utilizes a max pooling of size 5 and a stride of 2.

Finally, the encoded signals for each scale are concatenated to obtain the encoded signal, as shown by the equation below:

(2) $$f_{i}^{\prime \prime }\left(x\right)=\sum_{i}T_{i}\left(f_{i}^{\prime }\left(x\right)\right),$$

where *T_i_*(.) represents the *i*th encoding of the convoluted signal *f_i_*′(x) and Σ represents the concatenation of these encoded signals.

### 3.2. MsRes Structure

**Figure 2:**
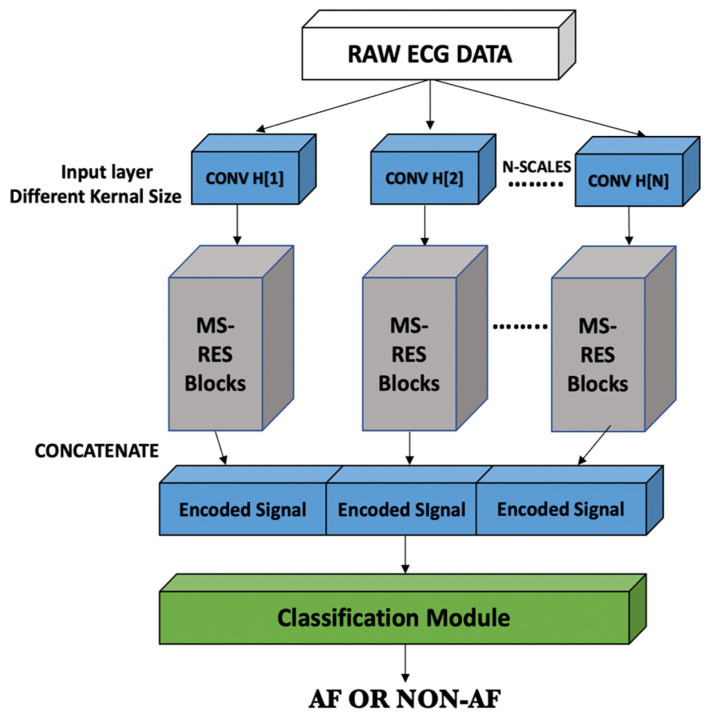
The proposed network architecture of the MsRes.

As shown in Figure 2, the architecture of the MsRes consists of several parallel convolutional neural networks, each of which learns features from a scale with a different kernel size. The parallel residual convolutional neural blocks learn their features and dependencies in different kernel sizes for each subnetwork. This process not only enriches and improves the feature detection, but also reduces the time needed for the completion of the process, since multiple networks are working in parallel; in addition, reduced resources are needed for the model. The networks can also grow in breadth rather than depth by increasing the usage of the layers that are already built, rather than adding hundreds of layers.

The model primarily consists of a convolutional layer as an input layer for the different streams. For multiscale encoding of the signal, first, the signal will convolve with different convolution kernels, as shown in Figure 2, with different combinations of kernel sizes of 3, 5, 7, 9, 11, and 13 assigned to each stream, with stride of 3. Each stream comprises seven consequent residual blocks for a different kernel size of the input for each stream. The output of these networks is concatenated to form the final encoded signal, which is used as the input of the classification module to predict the classes.

**Figure 3:**
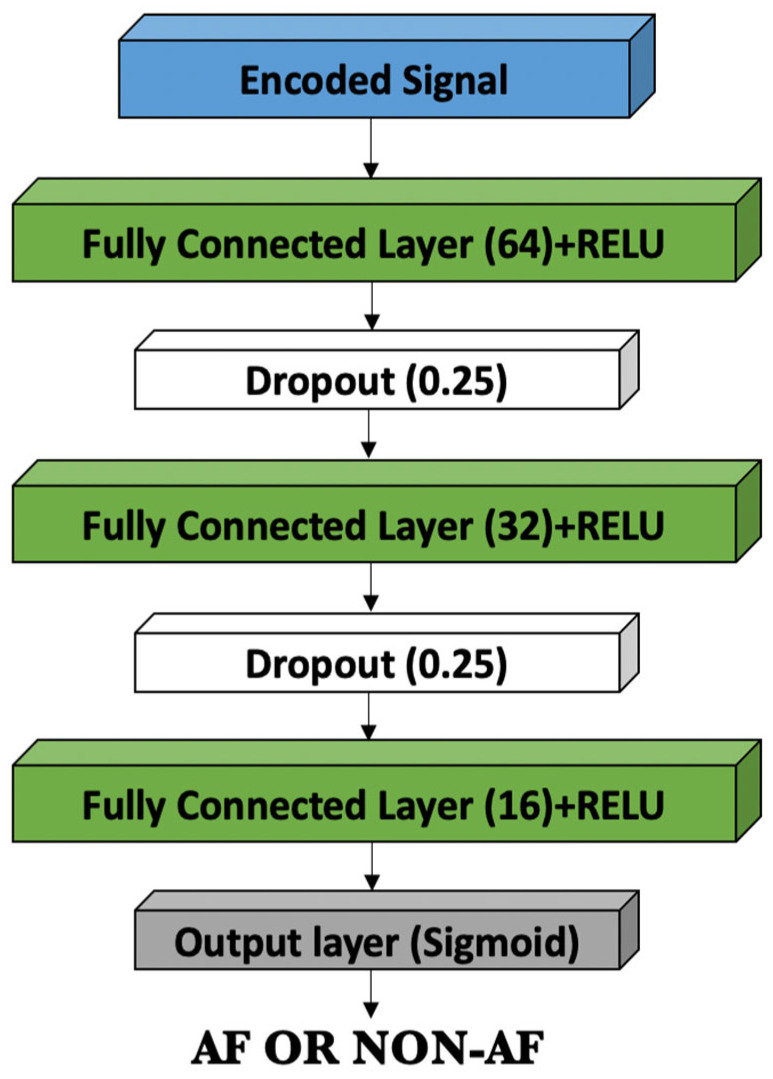
The classification module of the MsRes.

The classification module shown in Figure 3 consists of three dense layers with 64, 32, and 16 neurons and two dropout layers with (0.25). The dropout layers are employed between two dense layers to avoid and reduce overfitting. During training, the dropout layer selects a percentage of neurons at random and updates only the weights of the remaining neurons [52]. The dropout parameter was set to 0.25, which means that one-fourth of the neurons would not be updated to minimize the classification error and the network overfitting. In this model, we chose cross-entropy as a loss function. The output from the last dense layer was fed into a single sigmoid neuron, which outputs the predicted probability distribution over the two classes. Network complexity can be defined by the total number of training parameters, which depends on the number of scales used. Table 3 shows the parameters of a three-scale network that was optimized empirically, as discussed in Section 5. Here, the parameters for non-trainable layers, such as the pooling layer, dropout layer, and flatten layer, were excluded. The best-performing network has approximately 158,401 trainable parameters, which is considered a small number compared to the millions of parameters used by the models in the literature.

## 4. Experiments

**Figure 4:**
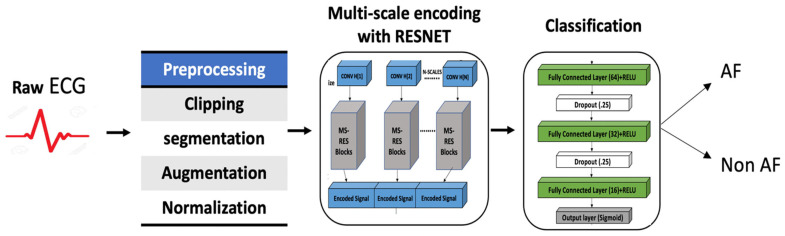
The workflow of the proposed atrial fibrillation (AF) detection method.

Generally, a deep-learning-based method consists of three main steps: preprocessing, feature representation, and classification. Following this baseline, in our method, a sequence of preprocessing steps was used—namely, signal clipping, segmentation, augmentation, and normalization—before the data were fed into the MsRes network for the encoding of the signal and the encoded signal was finally used for classification, as shown in Figure 4. Each record of ECG signals was segmented into windows of a certain time duration. The resulting data segments were divided into training and validation sets. The training data were augmented and then normalized before they were fed into the model for training. Finally, the test data were classified into one of two classes—AF or non-AF. These operations will be discussed in the following subsections in greater detail.

### 4.1. Data Used

The proposed method was evaluated using the PhysioNet Challenge 2017 dataset [28], which encourages the development of algorithms for classification from a single-lead short ECG recording in real conditions. Other existing databases (e.g., MIT-BIH AF database [4]) generally consist of cleaner data, and good performance can be obtained even by using simpler methods, such as feature thresholding. However, the PhysioNet Challenge 2017 dataset provides an opportunity to reliably detect AF in more real conditions, where many non-AF rhythms exhibit irregular RR intervals that may be similar to those in AF. Moreover, in this database, the infrequent occurrence of AF represents a real-life situation, and at the same time, this data imbalance becomes another challenge for the reliable detection of arrhythmias.

**Figure 5:**
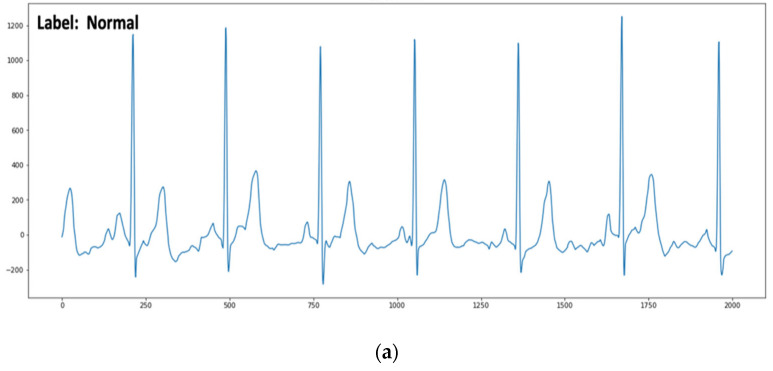

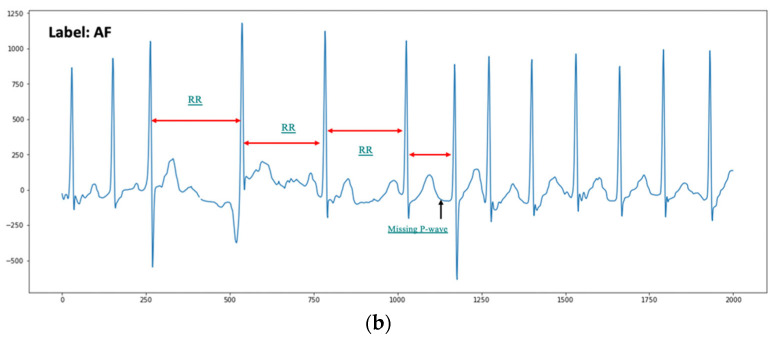
Examples of (**a**) normal and (**b**) AF ECG signals with irregular RR intervals and the absence of P-waves.

The PhysioNet CinC Challenge 2017 [28] database comprises 8528 ECG records that were captured by Alivecor’s portable single-channel ECG device and sampled at 300 Hz. The records’ lengths range from 9 to 61 s. The dataset comprises four types of ECG records—771 AF; 5154 normal (normal sinus rhythm); 46 noisy (too noisy to be recognized); 2557 other (other rhythms), as annotated by experienced experts. To transform the experiment into a binary classification problem, the ECG data that were annotated as normal, noisy, and other arrhythmias were labeled as non-AF. Thus, the dataset consisted of 771 records labeled as AF and 7757 records labeled as non-AF. Figure 5 shows examples of ECG signals belonging to the normal and AF classes. Table 4 describes the dataset’s statistics, including the number of records and time durations.

### 4.2. Signal Clipping

ECG data are frequently tainted by various noises and artifacts, which have an impact on the subsequent experiments and classification outcomes. Noise reduction can boost the effectiveness of a detection technique, especially for neural networks, whose performance is heavily reliant on the quality of the input data. It remains essential to adopt a reasonable method of preprocessing the data rather than applying rigorous preprocessing, which may result in the loss of viable information. In the dataset, some signals had serious noise disruptions at the start, which were primarily caused by noise when mounting the sensors or by the movements of the subjects. To remove this noise, the start of the signal was clipped for the removal of the affected part. In this study, the ECG signals were not filtered in the preprocessing stage for two reasons. First, the use of the original data reduced the computation cost. Second, the proposed model showed good performance with no filtering scheme, showing potential for use in practical applications.

### 4.3. Segmentation

In the dataset, the input ECG records have varying lengths, from 9 to 61 s. Inputs with varying lengths cannot be fit into CNN models. A consistent data length is necessary to make sure that the data can be entered into the model. For reliable detection of AF, we considered the use of a window of an ECG signal that adequately reflected the characteristics of the class that it belonged to. Although a short window was preferable for processing efficiency, a longer window could contain better diagnostic features. Several existing methods used a 8 to10 s window for reasonable performance in AF detection [19,20].

In this study, the experimental data were segmented into nine-second segments, which seems reasonable for the analysis of ECG rhythms with AF conditions and for the simplification of the computations. Because the sampling rate of the experimental data was at 300 Hz, 2700 samples were taken as a segment, with a moving window with a step size of 9. Hence, the number of samples (instances) increased. After the data segmentation, the number of samples became 27,108, containing both classes of which 24,729 were non-AF and 2379 were AF segments. To verify the influence of different input sizes on the model performance, the experimental data were also segmented into five- and ten-second segments.

### 4.4. Augmentation

**Figure 6:**
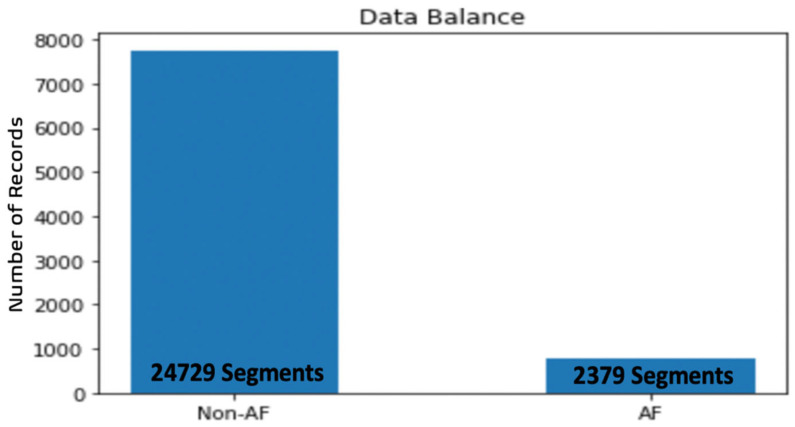
Distribution of segments of the two classes after segmentation.

As shown in Figure 6, the numbers of samples obtained in the two categories as a result of segmentation were severely unbalanced; there were far fewer AF samples than non-AF samples. If 80% of the segments were used for training, the training set included 21,686 segments, which would include 19,804 non-AF and 1882 AF segments. This unbalance dataset seriously affected the performance of the model and made it much trickier to screen an AF patient than a normal case. To solve this, oversampling was performed on the dataset, then the dataset was randomly divided into a training set and a test set at a proportion of 8:2. Because random oversampling might lead to overfitting, more sophisticated methods could be used, such as the synthetic minority oversampling technique (SMOTE) [53], an oversampling method that is commonly adopted and is based on finding the data’s closest neighbors by applying the Euclidean distance. By multiplying the nearest neighbors by a vector with values between 0 and 1, SMOTE can generate synthetic data [54]. There are several variations of SMOTE. Borderline–SMOTE (BLSMOTE) [55] oversamples only the minority examples near the borderline of the data. Adaptive synthetic (ADYSAN) [56], another well-known technique, is an improved version of SMOTE. After creating the samples, it adds a small random value so that, instead of all the samples being linearly correlated to the parent, they have a little more variance in them, that is, they are scattered.

We applied these augmentation techniques to the dataset to evaluate and investigate their effects on the model’s accuracy. Table 5 shows results of several experiments that were conducted for the comparison of these techniques. The experiments were performed using the network with three scales, and the performance is presented in terms of the F_1_ score and execution time; we observed that SMOTE outperformed the other two techniques. The default parameters of the SMOTE algorithm were used (sampling strategy = 1), which increased the samples in the minority class to equate with the size of the majority class. After balancing, there were a total of 39,608 segments in the training set.

### 4.5. Normalization

There was a significant fluctuation in the amplitudes of the ECG recordings between different records, as these records came from different people or even the same person with varying lead placements. It was discovered that in real-world scenarios, the neural network models converged more effectively when all of the inputs had similar ranges. Thus, amplitude of each of the ECG segments was normalized between 0 and 1 because normalizing the data also accelerated the speed of the model training.

### 4.6. Training and Classification

To ensure that the model would be fairly evaluated on the dataset, K-fold cross-validation (CV) was used; this is the most popular method in various DL applications. The original dataset was randomly divided into five subsets of equal size, and a five-fold cross-validation was used. The model’s average performance across the entire dataset was then estimated using the classification results of these five models.

#### Selection of Hyperparameters

To design an optimal structure for AF classification, we analyzed the impacts of different combinations of the hyperparameters based on experience and a manual random search technique [57], which was reported to be more efficient for hyperparameter optimization than a traditional grid search. Because we had a good number of hyperparameters, we chose the most important parameters empirically to balance the model performance with the number of training parameters and the training time. The hyperparameters that we chose included the number of residual blocks, the dropout percentage, the batch size, the learning rate, and the convolutional kernel size.

Seven residual blocks were chosen in each of the parallel MsRes blocks. The model was obtained by training with adaptive moment estimation (Adam) [58], which is an adaptation of the stochastic gradient descent (SGD) optimization algorithm. By minimizing the loss function, which represents the difference between the network prediction and the ground truth, Adam used the BP approach to determine the neural network’s parameter configuration. Adam is a method for computing adaptive learning rates that determines individual learning rates for various factors. The default learning rate is 0.001 [59]. A model typically learns more quickly when the learning rate is high and more steadily when the learning rate is low.

Different learning rates were explored, such as 0.001, 0.0001, and 0.005. It was found that the training process was more stable when the value of 0.001 was chosen. Additionally, we explored the use of different optimizers, such as Adadelta [60] and Adagrad [61], to see their impacts on the performance, but there was a drop in the accuracy and F_1_ score when optimizers other than Adam were used. The batch sizes were compared for a range of values (128, 256, and 512), and it was noticed that when the batch size was changed to 256 and 512, there was a negative impact on the performance accuracy and F_1_ score. The best performance was obtained with a value of 128.

The number of scales and the kernel sizes of each stream have a crucial impact on the feature detection performance of DL networks. We conducted our experiment to understand the effects of different signal encoding values on the feature detection in the data at hand and understand the effects of the different kernel sizes on the feature representation and model performance. The kernel size values were 3, 5, 7, 9, 11, and 13, and different combinations of these values were explored and applied to different numbers of streams, starting from two streams and going up to five. The best performance was obtained with three streams with kernel sizes of 5, 7, and 9.

Between two dense layers, a dropout layer was also used to reduce overfitting. During training, the dropout layer randomly selected a subset of neurons and only updated the weights of these neurons [52]. We set the dropout parameter to 0.25, which meant that one-fourth of the neurons would not be updated. Table 6 shows the values of the hyperparameters used for the MsRes to obtain the best performance in terms of accuracy and F_1_.

**Figure 7:**
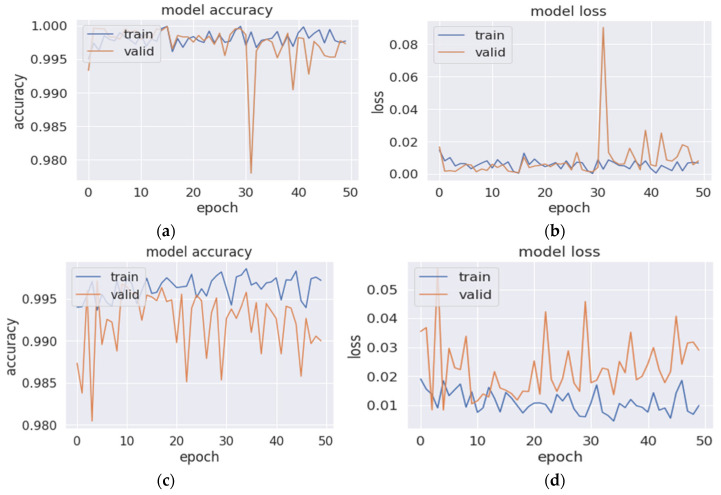
The accuracy and loss curves of the proposed model during the data training and validation (**a**,**b**) for the best-performing model with three scales and kernel sizes of 5, 7, and 9 and (**c**,**d**) for the model with four scales of 5, 7, 9, and 11.

The model loss was calculated using a binary cross-entropy function, which is preferable for binary classification. The loss was calculated according to the following formula:

(3) $$L=-\frac{1}{output\;size\;}\;\sum_{i=1}^{output\;size\;}\;Y_{i\;}\log\;\hat{Y_{i\;}}+(1-Y_{i\;})\;\log\;(1-\hat{Y_{i\;}{}_{\;}})$$

where *y* represents the expected outcome, and $\hat{y}$ represents the outcome produced by our model. Figure 7 shows the training and validation accuracy and loss curves of the proposed model. The first row in the figure shows the accuracy and loss curves for the best performance, with three streams with kernel sizes of 5, 7, and 9 over 50 epochs. They show that the training curves are relatively stable, and the presence of the fluctuations in the validation curves is due to the use of batch normalization, which may lead to unstable training and validation [62]. Furthermore, there is no degradation in the accuracy; instead, there is a decrease in the model loss and a gap between the two curves, which indicates a good fit and no overfitting. In the second row in the figure, the accuracy and loss curves for the use of four streams with kernel sizes of 5, 7, 9, and 11 show the instability of the training and validation curves, which affected the overall performance and degraded the F_1_ score. Such instability in the curve and the presence of the pumps are strong indications of what is called the overfit curve, which means that there were overfitting signs in the model when more than three streams were used.

## 5. Results and Analysis

In this section, the evaluation metrics, experimental results, and analyses and comparisons of the results are presented to explain the significance of our proposed multiscale signal encoding scheme.

### 5.1. Evaluation Metrics

The proposed system classifies the input ECG segments into one of two categories. We evaluated the classification performance using the *F*_1_ score as follows:

(4) $$F_{1}(\%)=\left(\frac{2\times Precision\times Recall}{Precision+Recall}\right)\;\times \;100\%,$$

Precision is defined as the ratio of true-positive (TP) classifications to the number of TP and false-positive (FP) classifications, and recall is the ratio of TP classifications to the number of TP and false-negative (FN) classifications. The following formulas were used to calculate the precision, recall, and accuracy:

(5) $$\mathrm{Precision}=\frac{\mathrm{TP}}{TP+FP}$$

(6) $$\mathrm{Recall}=\frac{\mathrm{TP}}{TP+FN}$$

(7) $$\mathrm{Accuracy}=\frac{\mathrm{TP}+\mathrm{TN}}{TP+TN+FP+FN}$$

**Figure 8:**
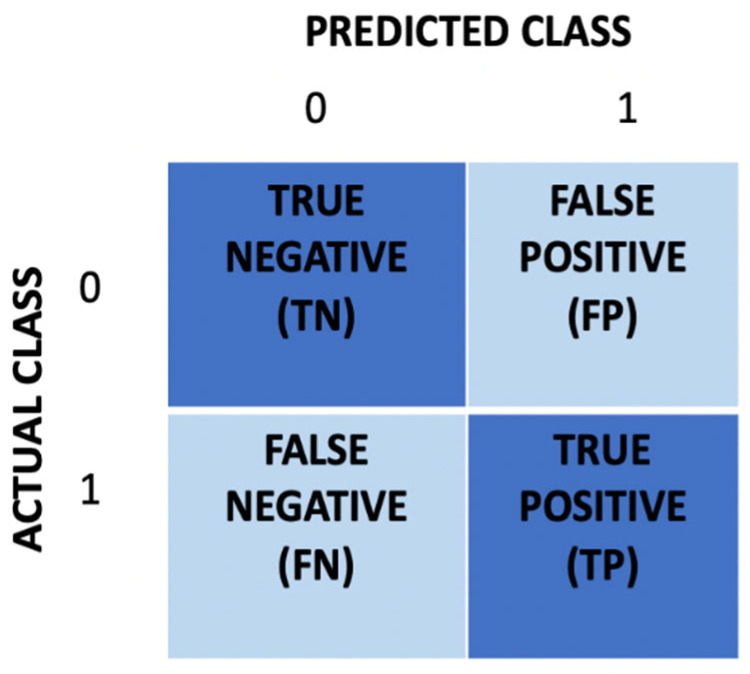
Confusion matrix for two classes.

The confusion matrix, which depicts the performance of a classification algorithm, can be used to calculate precision and recall. The matrix’s rows represent predicted classes, and its columns represent ground-truth classes. Figure 8 depicts a confusion matrix for two classes.

Accuracy is used in most cases for the evaluation of the classification performance. However, because there are more data in the positive class, accuracy is insufficient for evaluating the performance of an unbalanced dataset. Alternatively, we used the F_1_ score as a measure because it balances precision and recall and is useful when dealing with uneven class distributions [63].

### 5.2. Classification Performance

The proposed model was implemented in Google’s Colab environment with the Keras deep learning library using Python. Table 7, Table 8, Table 9, Table 10 and Table 11 show the experimental results for the multiscale encoding using different numbers of scales with different kernel sizes (i.e., 3, 5, 7, 9, 11, and 13). In Table 7, we present the experiment results with the single-stream kernel with different sizes. The best performance for the single scale was achieved with the kernel size of 13, which had an F_1_ score of 95.95%. The results of the use of two-stream multiscale encoding are shown in Table 8. A significant improvement was achieved, with the best performance showing an F_1_ score of 98.37% for kernel sizes of 5 and 7. The performance improved by 2–3% after the second scale was added. Subsequently, as shown in Table 9, the performance slightly improved with the addition of the third stream, and the highest average F_1_ score of 98.54% was achieved with three scales at the kernel sizes of 5, 7, and 9. The performance (best results obtained among alternative models) slightly dropped to 98.47% after the fourth stream was added and to 98.42% after the fifth stream was added, as shown in Table 10 and Table 11, respectively.

**Figure 9:**
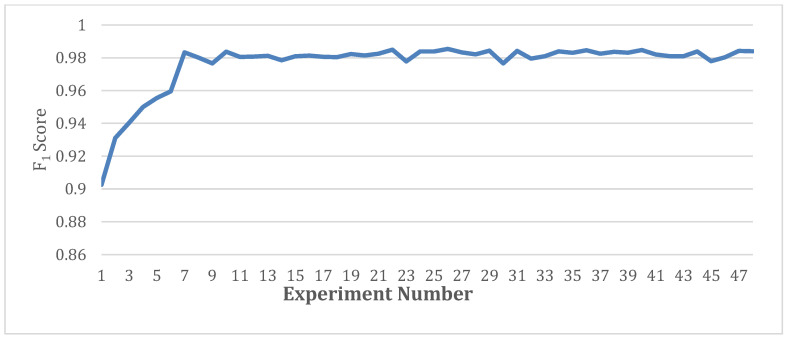
Performance curves showing the results of 48 experiments together (Table 7, Table 8, Table 9, Table 10 and Table 11) at different scales, sizes, and streams.

The fusion of information from the three scales (5, 7, and 9) generated the highest average F_1_ score of 98.54%, accuracy of 98.37%, precision of 97.75%, and recall of 98.67% for the model. These improved performance metrics can be attributed to the good capture of the critical diagnostic information, including the irregular RR intervals and P-wave abnormalities for this configuration. Adding bigger kernel values would cause the model to be disrupted with additional non-required information. Furthermore, it was noticed that using more than three streams did not improve performance, and it was inferred that this may have been due to the overfitting problem. These results validate the ability of the signal encoding of MsRes to capture clinically relevant information for improved AF diagnosis. Figure 9 shows the performance curve, representing the results of the 48 experiments in Table 7, Table 8, Table 9, Table 10 and Table 11 together according to the number of scales, sizes, and streams.

**Figure 10:**
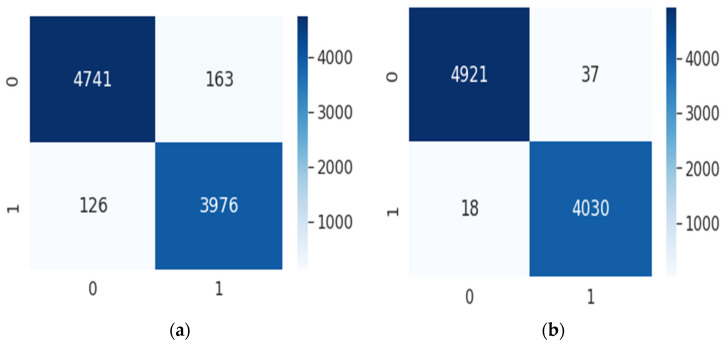

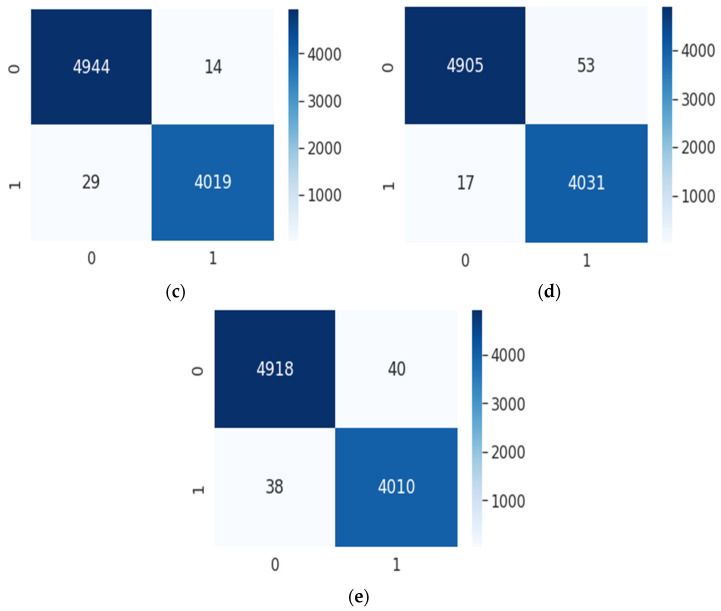
The proposed model’s confusion matrixes for the different numbers of scales: (**a**) single scale, (**b**) two scales, (**c**) three scales, (**d**) four scales, and (**e**) five scales.

Figure 10 shows five confusion matrices for each number of scales with the best performance combination for each scale according to the experimental results presented in Table 7, Table 8, Table 9, Table 10 and Table 11. The confusion matrix for five folds is shown with 0 indicating the case of non-AF and 1 indicating the case of AF. The diagonal elements show the number of correctly predicted records for each class. The off diagonals display the number of misclassifications for each class. In Figure 10a, the confusion matrix for the single scale with the kernel size of 13 is shown, and it indicates that 163 AF records were misclassified as non-AF and 126 non-AF records were misclassified as AF. The performance significantly improved, as seen in the confusion matrix for the two scales in Figure 10b, where only 37 AF records were misclassified as non-AF and 18 non-AF records were misclassified as AF. The performance further improved with the addition of the third scale in Figure 10c, wherein 14 AF records were misclassified as non-AF and 29 non-AF records were misclassified as AF. In the fourth and fifth scales, as shown in Figure 10d,e, the performance slightly dropped, and the number of misclassified AF records slightly increased.

**Figure 11:**
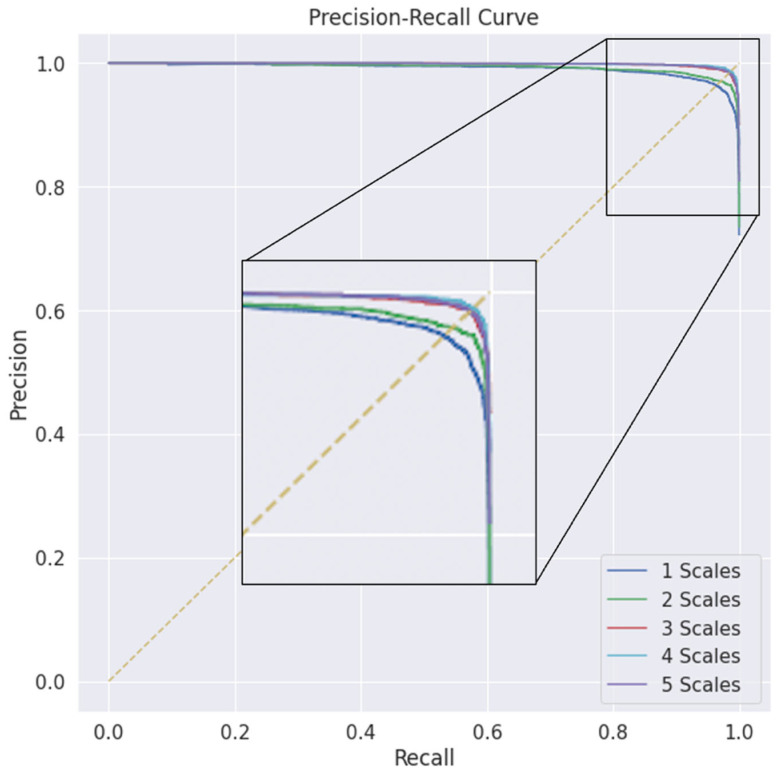
The precision–recall curves at different scales and kernel sizes.

**Figure 12:**
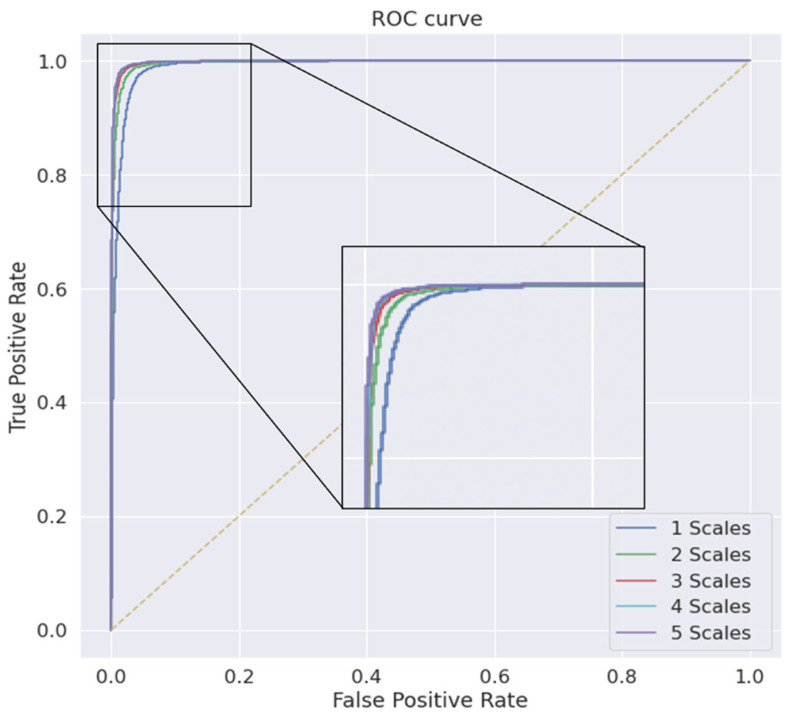
The proposed model’s receiver operating characteristic at different scales and kernel sizes.

Figure 11 shows the precision–recall curve, which indicates the tradeoff between the precision and recall for each number of scales. A larger area under the curve (AUC) indicates both higher recall and higher precision, where higher precision corresponds to a lower false-positive rate and higher recall corresponds to a lower false-negative rate. In the figure, the smallest AUC of the performance is shown with the single scale; a clear improvement in the AUC is shown when the number of scales increases to two and three; there is no clear line between the last three scales, and the performance waves of the third, fourth, and fifth scales are almost optimal, approaching the far-right corner. Similarly, Figure 12 shows the receiver operating characteristic (ROC) curve, which is the plot used for the representation of the diagnostic ability of binary classifiers in the same manner as that for different scales; there were clear improvements in the classification ability when the multiscale encoding scheme was used.

#### Experiments with Different Signal Lengths

To demonstrate the effectiveness of our AF detection system, we compared the performance of the best model (kernel sizes of 5, 7, and 9) for different signal lengths of five, nine, and ten seconds, as shown in Table 12. The best result was achieved with a signal length of nine seconds, which was the segment size that represented enough features to boost the detection. Our comparison of the five- and ten-second variations showed that a five-seconds window slightly reduced the performance in AF detection, whereas a ten-second window increased the computation complexity and the time without improving the detection performance.

### 5.3. Comparison with the Literature

Table 13 compares our proposed method with state-of-the-art methods in terms of the method, number of classes, input length, preprocessing, balancing, and F_1_ score. All of these methods were evaluated with the same unbalanced PhysioNet Challenge 2017 dataset. The two systems proposed in [64] and [3], which used a single-scale ResNet, had an average performance of 85% and 88%, respectively. The other three models using multiscale DL networks had an average performance of 85%, 92.1%, and 84.31%. These results show that our proposed method outperformed the state-of-the-art methods that were evaluated on the same database, with an F_1_ score of 98.54%.

Table 14 compares the different methods that were evaluated on the same dataset in terms of method, the number of classes, input length, preprocessing, balancing, trainable parameters, and F_1_ score. It shows that our proposed model has the lowest number of trainable parameters, which makes it reliable and applicable in real-time applications.

## 6. Conclusions

In this paper, we proposed a multiscale encoding scheme with residual blocks of deep CNNs—named MsRes—for screening out AF using short single-lead ECG signals. The proposed MsRes network applied the concept of multiscale convolutional signal encoding, which utilized different kernel sizes to capture the features at different scales of the input ECG recordings. The proposed system adopted a simple yet effective preprocessing scheme to produce a cropped and balanced number of segments for both classes without rigorous preprocessing and transformation. In the experiments that we conducted, we explored the effects of adding different numbers of scales with combinations of kernel sizes, which had a clear impact on the AF detection accuracy and F_1_ score.

Through an exhaustive evaluation, we found that the proposed MsRes method performed better in terms of the F_1_ score than the single-scale convolution kernel in several state-of-the-art methods evaluated with the PhysioNet Challenge 2017 ECG dataset, with an F_1_ score of 98.54%, an accuracy of 98.37%, a total loss of 5.6%, a precision of 97.75%, and a recall of 98.67%, when three multiscale encoding schemes with kernel sizes of 5, 7, and 9 were used. Additionally, the proposed method significantly reduced the network complexity, with 158,401 trainable parameters compared to the millions of parameters used in the literature.

One of the limitations of this work is that it was only tested on the PhysioNet Challenge 2017 ECG dataset. In the future, we will focus on improving MsRes, exploring the potential for further assessment of its performance and generalization to other public databases, or using cross-databases to further improve and optimize its learning ability. Another limitation is that our model was designed only for a two-class classification problem, namely, that of AF and non-AF detection. We would like to extend our method to a multiclass classification problem in which a wide variety of different arrhythmias could be detected from single-lead ECG signals to confirm a high diagnostic output close to that of cardiologists.

## Figures and Tables

**Table 3:** The proposed detailed structure of the system describes the input of each layer with the number of parameters.

	Output Shape	# Parameters	Output Shape	# Parameters	Output Shape	# Parameters	Activation
**INPUT LAYER**	(2700, 1)	0					
	**STREAM 1(5)**		**STREAM 2(7)**		**STREAM 3(9)**		
**CONV1D**	(900, 32)	192	(899, 32)	256	(898, 32)	320	
**CONV1D**	(900, 32)	3104	(899, 32)	3104	(898, 32)	3104	**ReLU**
**CONV1D**	(900, 32)	3104	(899, 32)	3104	(898, 32)	3104	**ReLU**
**ADD**	(900, 32)	0	(899, 32)	0	(898, 32)	0	
**MaxPooling1D**	(498, 32)	0	(497, 32)	0	(497, 32)	0	
**CONV1D**	(498, 32)	3104	(497, 32)	3104	(497, 32)	3104	**ReLU**
**CONV1D**	(498, 32)	3104	(497, 32)	3104	(497, 32)	3104	**ReLU**
**ADD**	(498, 32)	0	(497, 32)	0	(497, 32)	0	
**MaxPooling1D**	(247, 32)	0	(247, 32)	0	(247, 32)	0	
**CONV1D**	(247, 32)	3104	(247, 32)	3104	(247, 32)	3104	**ReLU**
**CONV1D**	(247, 32)	3104	(247, 32)	3104	(247, 32)	3104	**ReLU**
**ADD**	(247, 32)	0	(247, 32)	0	(247, 32)	0	
**MaxPooling1D**	(122, 32)	0	(122, 32)	0	(122, 32)	0	
**CONV1D**	(122, 32)	3104	(122, 32)	3104	(122, 32)	3104	**ReLU**
**CONV1D**	(122, 32)	3104	(122, 32)	3104	(122, 32)	3104	**ReLU**
**ADD**	(122, 32)	0	(122, 32)	0	(122, 32)	0	
**MaxPooling1D**	(59, 32)	0	(59, 32)	0	(59, 32)	0	
**CONV1D**	(59, 32)	3104	(59, 32)	3104	(59, 32)	3104	**ReLU**
**CONV1D**	(59, 32)	3104	(59, 32)	3104	(59, 32)	3104	**ReLU**
**ADD**	(59, 32)	0	(59, 32)	0	(59, 32)	0	
**MaxPooling1D**	(28, 32)	0	(28, 32)	0	(28, 32)	0	
**CONV1D**	(28, 32)	3104	(28, 32)	3104	(28, 32)	3104	**ReLU**
**CONV1D**	(28, 32)	3104	(28, 32)	3104	(28, 32)	3104	**ReLU**
**ADD**	(28, 32)	0	(28, 32)	0	(28, 32)	0	
**MaxPooling1D**	(12, 32)	0	(12, 32)	0	(12, 32)	0	
**CONV1D**	(12, 32)	3104	(12, 32)	3104	(12, 32)	3104	**ReLU**
**CONV1D**	(12, 32)	3104	(12, 32)	3104	(12, 32)	3104	**ReLU**
**ADD**	(12, 32)	0	(12, 32)	0	(12, 32)	0	
**MaxPooling1D**	(4, 32)	0	(4, 32)	0	(4, 32)	0	
**CONCATENATE**	(4, 96)	0					
**FLATTEN**			384	0			
**DENSE + Dropout**	64	24,640					
**DENSE + Dropout**	32	2080					
**DENSE**	16	528					
**DENSE** **Total parameters**	1 **158,401**	17					**Sigmoid**

**Table 4:** Statistics of the PhysioNet Challenge 2017 dataset.

Label	New Label	Records	Time	
			Min	Max
**AF**	AF	771	10.0	60
**Normal**	Non-AF	5154	9.0	61.0
**Noisy**		46	10.2	60
**Other**		2557	9.1	60.9
**Total**		8528		

**Table 5:** Comparison of the effects of different balancing techniques on the model performance.

Technique	Execution time	Precision %	Recall %	Accuracy %	F_1_ %
**Without balancing**	-	88.01	83.65	97.54	85.71
**ADYSAN**	6 s	97.45	96.85	97.41	97.14
**Borderline-SMOTE**	6 s	97.79	98.27	98.21	98.02
**SMOTE**	1 s	97.75	98.67	98.37	**98.54**

**Table 6:** The selected parameters of the MsRes.

Hyperparameter	Optimal Value
Number of residual blocks in an MsRes block	7
Learning rate	0.001
Batch size	128
Dropout rate	0.25

**Table 7:** Classification performance of the model for a single scale with different kernel sizes.

Experiment No.	Kernel Size	Precision	Recall	Accuracy	F_1_	Total Parameters
**1**	3	0.9277	0.8704	0.8943	0.9026	6577
**2**	5	0.9484	0.9151	0.9302	0.9311	9589
**3**	7	0.9588	0.9231	0.9393	0.9404	12,937
**4**	9	0.9642	0.9366	0.9492	0.9500	16,621
**5**	11	0.9774	0.9347	0.9544	0.9555	20,641
**6**	**13**	**0.9673**	**0.9520**	**0.9592**	**0.9595**	**24,997**

**Table 8:** Classification performance of the model for two scales with different kernel sizes.

Experiment No.	Kernel Size	Precision	Recall	Accuracy	F_1_	Total Parameters
**7**	3, 5	0.9746	0.9921	0.9847	0.9833	106,305
**8**	3, 7	0.9789	0.9878	0.9857	0.9801	106,369
**9**	3, 9	0.9661	0.9878	0.9785	0.9766	106,433
**10**	3, 11	0.9734	0.9878	0.9823	0.9805	106,497
**11**	3, 13	0.9747	0.9829	0.9807	0.9807	106,561
**12**	**5, 7**	**0.9808**	**0.9867**	**0.9852**	**0.9837**	**106,433**
**13**	5, 9	0.9708	0.9921	0.9828	0.9812	106,497
**14**	5, 11	0.9782	0.9790	0.9806	0.9785	106,561
**15**	5, 13	0.9810	0.9809	0.9828	0.9809	106,625
**16**	7, 9	0.9793	0.9835	0.9831	0.9813	106,561
**17**	7, 11	0.9705	0.9916	0.9821	0.9806	106,625
**18**	7, 13	0.9715	0.9897	0.9820	0.9804	106,689
**19**	9, 11	0.9801	0.9845	0.9840	0.9823	106,689
**20**	9, 13	0.9780	0.9850	0.9832	0.9814	106,753

**Table 9:** Classification performance of the model for three scales with different kernel sizes.

Experiment No.	Kernel Size	Precision	Recall	Accuracy	F_1_	Total Parameters
**21**	3, 5, 7	0.9775	0.9878	0.9841	0.9825	158,209
**22**	3, 5, 9	0.9806	0.9894	0.9863	0.9849	158,273
**23**	3, 5, 11	0.9744	0.9837	0.9808	0.9838	158,337
**24**	3, 5, 13	0.9751	0.9892	0.9837	0.9821	158,401
**25**	3, 7, 9	0.9776	0.9784	0.9799	0.9778	158,337
**26**	3, 7, 11	0.9791	0.9886	0.9852	0.9838	158,401
**27**	3, 7, 13	0.9792	0.9877	0.9849	0.9833	158,465
**28**	3, 9, 11	0.9796	0.9892	0.9858	0.9843	158,465
**29**	3, 9, 13	0.9775	0.9910	0.9856	0.9842	158,529
**30**	**5, 7, 9**	**0.9775**	**0.9867**	**0.9837**	**0.9854**	**158,401**
**31**	5, 7, 11	0.9776	0.9759	0.9791	0.9766	158,465
**32**	5, 7, 13	0.9725	0.9867	0.9814	0.9795	158,529
**33**	5, 9, 11	0.9743	0.9878	0.9826	0.9809	158,529
**34**	5, 9, 13	0.9785	0.9894	0.9854	0.9839	158,593
**35**	7, 9, 11	0.9792	0.9870	0.9844	0.9830	158,593
**36**	7, 9, 13	0.9802	0.9891	0.9860	0.9846	158,657

**Table 10:** Classification performance of the model for four scales with different kernel sizes.

Experiment No.	Kernel Size	Precision	Recall	Accuracy	F_1_	Total Parameters
**37**	3, 5, 7, 9	0.9789	0.9864	0.9841	0.9825	210,177
**38**	3, 5, 7, 11	0.9835	0.9838	0.9853	0.9836	210,241
**39**	3, 5, 7, 13	0.9804	0.9858	0.9847	0.9831	210,305
**40**	**3, 5, 9, 11**	**0.9852**	**0.9843**	**0.9863**	**0.9847**	**210,305**
**41**	3, 7, 9, 11	0.9771	0.9871	0.9836	0.9820	210,369
**42**	5, 7, 9, 11	0.9770	0.9854	0.9827	0.9810	210,433

**Table 11:** Classification performance of the model for five scales with different kernel sizes.

Experiment No.	Kernel Size	Precision	Recall	Accuracy	F_1_	Total Parameters
**43**	3, 5, 7, 9, 11	0.9811	0.9810	0.9829	0.9809	262,209
**44**	3, 5, 7, 9, 13	0.9815	0.9862	0.9853	0.9838	262,273
**45**	3, 5, 7, 11, 13	0.9799	0.9763	0.9802	0.9779	262,337
**46**	3, 5, 9, 11, 13	0.9711	0.9896	0.9820	0.9803	262,401
**47**	**3, 7, 9, 11, 13**	**0.9791**	**0.9894**	**0.9842**	**0.9842**	**262,465**
**48**	5, 7, 9, 11, 13	0.9800	0.9880	0.9854	0.9840	262,529

**Table 12:** Comparison of the results of our proposed model with different signal lengths.

Signal Length	Precision %	Recall %	Accuracy %	F_1_ %
**5 s**	96.77	98.63	97.88	97.69
**9 s**	97.75	98.67	98.37	**98.54**
**10 s**	97.08	98.51	97.99	97.78

**Table 13:** Comparison of the results of our proposed model with those of existing state-of-the-art methods.

Reference	Method	Classes	Input Length	Preprocessing	Balancing	F_1_
**Jinjing Zhu et al.** [64] **2019**	94-layer ResNet	4	27 s	Filtering + R_−_ peak detection	Augmentation	85.00%
**Nankani et al.** [3] **2019**	16-layer Resnet	4	Different	One-hot encoding + Filtering	Down-sampling Augmentation	88.00%
**Ping et al.** [65] **2020**	Multiscale CNN and LSTM	2	5 s	-	Oversampling	85.00%
**CAO et al.** [66] **2019**	Multiscale ResNet Structure	4	9 s	DWT	Overlap Sampling	92.10%
**Prabhakararao et al.** [41] **2020**	Multiscale Spectrogram Convolution	2	5 s	Filtering + Transformation	Oversampling	84.31%
**Proposed 2022**	**Multiscale Residual Signal Encoding**	**2**	**9 s**	**-**	**SMOTE**	**98.54%**

**Table 14:** Comparison of the results of our proposed model with those of different models in terms of trainable parameters.

Reference	Method	Classes	Input Length	Preprocessing	Balancing	Trainable Parameters	F_1_
**Fernando et al.** [23]	ResNet	4	30 s	-	Augmentation	1,500,000	77.45%
**Martin et al.** [29]	CNN + LSTM	4	9 s	Transformation	Augmentation	530,210	82%
**Jonathan et al.** [67]	CNN + post-processing	4	15 s	Transformation	Augmentation	262,344	85%
**Proposed**	**Multiscale Residual Signal Encoding**	**2**	**9 s**	**-**	**SMOTE**	**158,401**	**98.54%**

## Data Availability

Databases used in this study are available online.
